# What’s the fuss about? Parent presentations of fussy eating to a parenting support helpline

**DOI:** 10.1017/S1368980017004049

**Published:** 2018-02-01

**Authors:** Holly A Harris, Bonnie Ria-Searle, Elena Jansen, Karen Thorpe

**Affiliations:** 1 Centre for Children’s Health Research, Queensland University of Technology, 62 Graham Street, South Brisbane, QLD 4101, Australia; 2 School of Exercise and Nutrition Sciences, Queensland University of Technology, Kelvin Grove, Brisbane, Australia; 3 Institute for Social Science Research, The University of Queensland, Indooroopilly, Australia

**Keywords:** Fussy eating, Picky eating, Children, Parental feeding, Telehealth

## Abstract

**Objective:**

To characterise parent presentations of fussy eating and mealtime interactions at a point of crisis, through analyses of real-time recordings of calls to a parenting helpline.

**Design:**

Qualitative analysis included an inductive thematic approach to examine clinical parent presentations of fussy eating and derive underlying themes relating to mealtime interactions.

**Setting:**

Calls made to the Child Health Line regarding feeding concerns were recorded and transcribed verbatim.

**Subjects:**

From a corpus of 723 calls made during a 4-week period in 2009, twelve were from parents of children aged 6–48 months.

**Results:**

Parents of infants (≤12 months, *n* 6) presented feeding concerns as learning challenges in the process of transitioning from a milk-based to a solid-based diet, while parents of toddlers (13–48 months, *n* 6) presented emotional accounts of feeding as an intractable problem. Parents presented their child’s eating behaviour as a battle (conflict), in which their children’s agency over limited intake and variety of foods (child control) was constructed as ‘bad’ or ‘wrong’. Escalating parent anxiety (parent concern) had evoked parent non-responsive feeding practices or provision of foods the child preferred.

**Conclusions:**

Real-time descriptions of young children’s fussy eating at a time of crisis that initiated parents’ call for help have captured the highly charged emotional underpinnings of mealtime interactions associated with fussy eating. Importantly, they show the child’s emerging assertion of food autonomy can escalate parents’ emotional distress that, in the short term, initiates non-responsive feeding practices. The current study identifies the importance of educational and emotional support for parents across the period of introducing solids.

Food preferences are established in childhood^(^ ^1^ ^)^. However, childhood is also a period in which foods associated with long-term health and prevention of chronic diseases, such as fruits and vegetables^(^ ^2^ ^)^, are often rejected^(^ ^3^ ^)^. Children who reject new or familiar foods are frequently referred to as ‘food neophobic’ or a ‘fussy/picky eater’^(^ ^4^ ^)^, although fussy eating is a relatively common and transient phase in childhood^(^ ^5^ ^)^. Despite the typically temporary nature of fussy eating, food rejection is a significant source of parental stress^(^ ^6^ ^)^ that may serve to perpetuate suboptimal nutrition and ongoing problematic eating behaviours. How parents respond to fussy eating may exacerbate the trajectory of fussy eating^(^ ^7^ ^)^. To understand the dynamics of fussy eating in the parent–child feeding relationship, the present paper utilises unique real-time data from recordings of all calls made by parents to a helpline in a 4-week period seeking support for feeding concerns related to fussy eating. The calls capture parents’ perspectives at a time of crisis that has initiated professional help-seeking and provide a rare opportunity to characterise feeding problems at the time these are experienced. The current study examines parent presentation of child fussy eating and their characterisation of mealtime interactions underlying fussy eating.

Definitions of fussy eating vary widely, due to measurement methodologies and the ages at which assessments are made^(^ ^8^ ^)^. Parent-reported assessments of fussy eating often refer to a child’s low quantity or variety of foods consumed, or strong food preferences^(^ ^6^ ^,^ ^9^ ^)^. Less common parent-reported assessments include the child’s slow eating and other ‘food avoidant’ eating behaviours^(^ ^3^ ^)^, selectivity of food presentation or preparation, low appetite^(^ ^10^ ^)^, disinterest or problematic behaviour during mealtimes^(^ ^11^ ^)^, gagging^(^ ^12^ ^)^ and texture aversions^(^ ^13^ ^)^. Although questionnaires are convenient tools to measure fussy eating at a population level^(^ ^8^ ^)^, predetermined questions limit the characterisation of children’s fussy eating to behaviour. Conversely, qualitative studies provide a contextual, first-hand account of how parents describe specific feeding concerns that can capture emotional content^(^ ^14^ ^)^.

Parental feeding concerns can manifest in anxiety that adversely impacts the child–parent relationship^(^ ^15^ ^)^. For example, mothers of children who are difficult to feed experience more negative emotions and use ‘non-responsive’ feeding practices, such as using liked food as a reward, pressuring and offering alternatives^(^ ^16^ ^)^. Through a dialectical lens of child and parent as equal agents^(^ ^17^ ^)^, strain is placed on the feeding relationship when discordance exists between child agency (expressing food preferences or autonomy to their parent) and parent agency (influencing their child’s food choice and consumption). Uncooperative interactions between child and parent are thought to result in child resistance and/or parents catering to the child’s preference^(^ ^18^ ^)^. Conversely, Satter’s division of responsibility in feeding highlights cooperative interactions, where agency is divided into ‘parent provides’ (what, where and when) and ‘child decides’ (how much, if any)^(^ ^19^ ^)^. Understanding parent perspectives of mealtimes and identifying uncooperative mealtime interactions could be key to improving feeding relationships.

Maternal child health nurses are often the first health professionals to provide primary intervention for parents. In Australia, the site of the current study, parents visit a child health nurse approximately fourteen times within the first 12 months of their child’s life, for developmental check-ups and parenting support^(^ ^20^ ^)^. Nurse telephone helplines provide health care and parenting support out-of-hours and for those with problems of access. These services are used universally^(^ ^21^ ^)^; however, research on child health nurse-staffed telephone helplines is limited. One study from the UK found that parents perceive nurse-staffed telephone helpline advice as trustworthy, empowering and reassuring; as well as affordable and convenient^(^ ^22^ ^)^. In a survey of Australian parents of toddlers seeking information regarding feeding problems, almost half had previously accessed a child health nurse, while one-quarter had called a nurse-staffed child health helpline^(^ ^23^ ^)^.

Definitions of fussy eating to date have generally relied upon parental report via questionnaire, and less frequently retrospective focus groups^(^ ^12^ ^)^ or interviews with parents^(^ ^9^ ^,^ ^14^ ^)^. The current research, an exploration of helpline calls made by parents about their young child’s feeding, gives a unique snapshot of parental perceptions at a crisis point. The aims of the current study were to: (i) describe how parents present child fussy eating at ‘crisis point’; and (ii) identify the nature of the feeding interactions described by the parent that has brought them to this point.

## Methods

### Study design

The current qualitative, cross-sectional research draws on data from the Calling for Help study, carried out in 2009^(^ ^24^ ^)^. Phone calls made to the Australian Child Health Line in Queensland, Australia were recorded over a period of 4 weeks. At the time of data collection, about 50 000 calls per year were taken by qualified nurses with additional midwifery or child health training. Within the original sample, 48 % of calls were made regarding parenting advice, 22 % were seeking medical advice and 26 % were medical and parenting issues combined^(^ ^24^ ^)^. While we acknowledge that the data were collected 8 years from the current secondary data analysis, we have no reason to believe that parents’ descriptions of fussy eating provided to the Child Health Line are likely to have changed since the data recording. Our analyses focus on parental descriptions and do not examine the advice provided to parents, which may evolved over time. The study protocol was approved by the Queensland University of Technology Human Research Ethics Committee and the Royal Brisbane Woman’s Hospital Ethics Committee. Prior to the calls being recorded, the child health nurses provided informed consent. The nurse could cease recording at any time during the call or if the nature of the call broke confidentiality. Participants provided informed verbal consent to the recording of the call for research purposes. Callers could refuse to the recording of the call, or withdraw from the call being recorded at any point in the conversation or at a later date. In the event, only one call was withdrawn by a parent whose content was focused on mental health. No nurses withdrew.

### Participants

Participants for the current study were parents who made phone calls to the Child Health Line seeking advice about ‘feeding concerns related to fussy eating’ in children aged from 6 to 48 months. This age group was purposefully selected as the focus for our study, as fussy eating has been shown to decline after the age of 4 years^(^ ^5^ ^,^ ^25^ ^)^. ‘Feeding concerns related to fussy eating’ were classified as parents expressing difficulties feeding solid foods, as opposed to breast milk or formula. To differentiate between ‘feeding concerns related to fussy eating’ and temporary reductions in appetite (i.e. due to the child being sick), only phone calls describing an ongoing problem were included. From a total of 723 calls screened, twelve (2 %) presented concerns related to fussy eating. Sociodemographic characteristics of the caller and child were recorded. These included caller (parent) gender, child age, current breast-feeding status and a postcode, which was linked to an index of socio-economic status as per the Socio-Economic Index for Areas^(^ ^26^ ^)^ (SEIFA; Table 1). SEIFA is measured on a scale of 1–10, with the highest value indicating greater relative advantage^(^ ^26^ ^)^. Participants were categorised into ‘low’ (1–3), ‘medium’ (4–7) and ‘high’ (8–10) socio-economic status based on their SEIFA score.

**Table 1:** Characteristics of parents (*n* 12) presenting concerns related to their child’s fussy eating to the Child Health Line in Queensland, Australia, over a 4-week period in 2009

Parent	Mother/father	Age of child (months)	Currently breast-feeding (Y/N)	SEIFA code	Socio-economic status*
1	Mother	30	N	8	High
2	Mother	48	N	5	Medium
3	Mother	10	N	10	High
4	Mother	24	N	10	High
5	Mother	12	N	3	Low
6	Mother	9	N	–	–
7	Mother	12	N	6	Medium
8	Father	22	N	10	High
9	Mother	10	N	3	Low
10	Mother	7	N	8	High
11	Mother	7	Y	9	High
12	Mother	36	N	3	Low

Y, yes; N, no; SEIFA, Socio-Economic Index for Areas (an index of socio-economic status measured on a scale of 1–10, with the higher values indicating greater relative advantage^(^ ^26^ ^)^); –, missing data.

* Socio-economic status categorised based on SEIFA scores: 1–3=‘low’, 4–7=‘medium’, 8–10=‘high’.

### Data analysis

The corpus of twelve calls were transcribed verbatim. Data analysis followed an inductive thematic approach as outlined by Braun and Clarke^(^ ^27^ ^)^. This involved a process of immersion within the data, progressive connection of coded ideas across the data and the refinement of emerging themes. Similarities in phrases and words used were noted using open coding. To address Aim 1, the opening lines of the conversation were analysed to examine how parents presented their concerns related to fussy eating. To address Aim 2, open coding was used to record emergent themes to capture the full range and depth of parental descriptions of feeding interactions. All authors contributed to analysis of data with codes and themes triangulated between the four authors.

## Results

The available sociodemographic data indicated that the callers were mostly mothers (11/12) and half were from a ‘high’ socio-economic area (6/12). A summary of the characterisitcs is presented in Table 1. Presentations of feeding concerns related to fussy eating were distinct for infants and toddlers. Illustrative quotes are presented in Table 2. The calls advanced to detail descriptions of mealtime interactions as a focus for conflict, with parent concern and the child’s control over food, feeding and family emerging as key themes.

**Table 2:** Parent (*n* 12) presentations of fussy eating to the Child Health Line, in Queensland, Australia, over a 4-week period in 2009, by infant and toddler group

Theme	Quote
Infant group (≤12-months, *n* 6)	
2a. Refusal of solids/textured foods	‘Oh he’s very hit and miss. Like sometimes he’ll have hardly a few spoons then he’ll start whingeing.’ (Parent 6; 9-month-old child)
	‘He’s just, he’s very much on milk, not having any solids at all.’ (Parent 7; 12-month-old child)
	‘ ... he doesn’t like anything that’s lumpy, or he’ll just hold it in his mouth and just vomit.’ (Parent 9; 10-month-old child)
	‘But I mean she’s not eating solid foods. She’s having trouble eating solid foods. Like she chokes on all hard foods.’ (Parent 10; 12-month-old child)
Toddler group (13–48-months, *n* 6)	
2b. Quantity of foods	‘He’s just one of those kids that won’t even try it.’ (Parent 2; 48-month-old child)
	‘Um, he just won’t try anything, he just won’t touch anything …’ (Parent 1; 30-month-old child)
	‘I have a 22-month-old daughter and she won’t eat.’ (Parent 8; 22-month-old child)
2c. Variety	‘Now he’s pretty much doesn’t eat anything and he has his bottles and he’ll ask for biccies [*sic*] but he just won’t have anything else.’ (Parent 1; 30-month-old child)
	‘He’s been a tiny eater, um, and when he does eat, it’s really bad, he won’t eat fruit, he won’t eat vegetables, he won’t eat potatoes, he won’t eat meat. He’s basically, his diet is generally all bread.’ (Parent 4; 24-month-old child)
	‘I’ll cut up a little square piece of bread with his dinner and that too and he won’t eat his food, normally he does and he’ll probably eat that little bit of bread but he’s eating more bread than he is anything else.’ (Parent 5; 12-month-old child)
	‘I mean she’ll eat, she’ll eat steak. That’s the only thing she’ll eat. She won’t touch vegetables at all.’ (Parent 8; 22-month-old child)
2d. Refusal of solids/textured foods	‘And she usually has a bottle and we kind of make her some breakfast. Sometimes she’ll eat her breakfast but [it’s] usually a yoghurt. She won’t ever have fruit or anything like that or cereal or anything. And then we will try and give her a snack but she’ll usually only have a biscuit or a vegemite sandwich or something like that if we’re lucky. Usually, mainly it’s just bottles and you know each time around twelve thirty she’ll sit down for lunch but she won’t, she won’t eat it. And then she’ll have a nap and then about five, five thirty we’ll try and give her dinner and dinner’s the worst of the lot.’ (Parent 8; 22-month-old child)

### Aim 1: Parent problem presentation

**Fig. 1:**
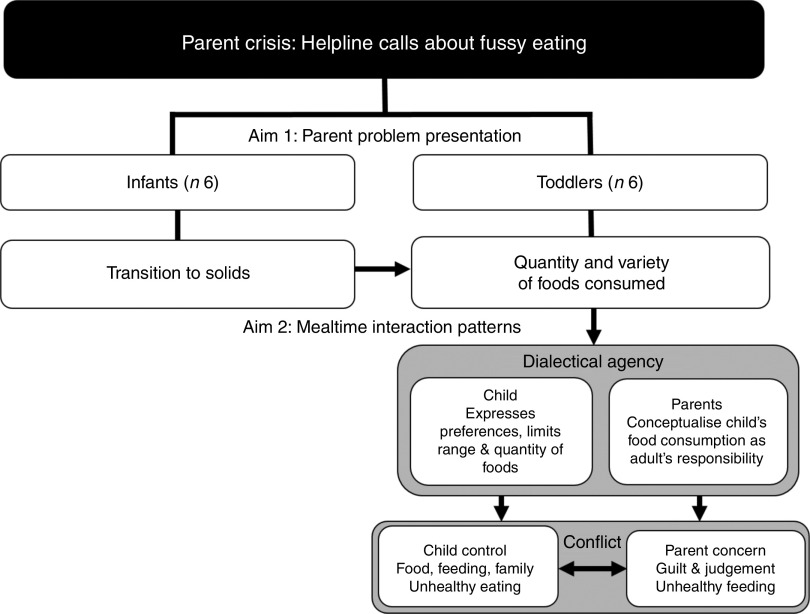
Flow diagram of parents (*n* 12) presenting concerns related to their child’s fussy eating and descriptions of mealtime interactions to the Child Help Line in Queensland, Australia, over a 4-week period in 2009. ‘Infants’ include parents of children aged 6–12 months; ‘toddlers’ include parents of children aged 12–48 months

Parents were separated into two main groups, based on their child’s age and the content of the call (see Fig. 1). The first group were parents of infants ≤12 months old (*n* 6), whereby parents’ descriptions centred around the infant’s refusal of solids or textured foods. The second group consisted of toddlers aged between 13 and 48 months (*n* 6), whose parents described greater detail and concern over mealtime behaviour and lack of variety and/or volume of foods. Due to the distinctive differences between these two groups, infant and toddler data were analysed separately to address Aim 1.

#### Infant group

Parents of children in the infant group presented challenges relating to transitioning to solid foods. Parents’ descriptions presented concern about the infant’s refusal of solid or textured food (Table 2, theme 2a). Notable in these descriptions is an emphasis on the problem as located with the child rather than as something that is a problem of parenting. That is, parents’ presentations are conceptualised as the child’s learning challenges in the process of transitioning to solids. This is expressed both as a positive conceptualisation of the child as ‘having trouble’ and as a negative conceptualisation of the child as ‘whingeing’. Perhaps as a result of this conceptual framing of the problem being outside parent responsibility in these calls, the parents generally do not go on to discuss the details of feeding but rather seek advice or strategies from the nurse call-taker.

#### Toddler group

Parents of children in the toddler group labelled their child as a ‘fussy eater’ and used high-intensity, dichotomous language inferring moral judgement to describe their child’s eating behaviours (i.e. using terms such as ‘good’/‘bad’, ‘wrong’/‘right’, ‘will’/‘not’). Generally, this description centred on the quantity of food the child consumed (Table 2, theme 2b). Notable in the quotes in Table 2, theme 2b is the parents’ use of future-oriented words such as ‘will not’ and the building conceptualisation of the child as defiant. In contrast to the descriptions provided for infants, we see parents presenting their child’s eating as a behavioural problem and the emergence of inference of intent. Parent concern about their child’s eating clearly emerges in the parents’ descriptions with an evident tension between the quantity of foods consumed and the variety and/or the nutritional quality of foods preferred (Table 2, theme 2c). Limited variety of food groups is a major emergent concern among the toddler group. Some parents described their child’s difficult eating habits as a function of lack of variety or balanced intake of food groups. Like descriptions provided by the parents of infants, parents of toddlers also mentioned their toddlers’ refusal of solids and aversion to texture (Table 2, theme 2d). However, there is an escalation of concern and frustration evidenced in more emotive language.

Unique to the parents in the toddler group are their descriptions of their child’s limited dietary variety – often related to certain food groups – and lower quantity of foods consumed than what was expected by the parent. These descriptions were largely conceptualised as negative. In contrast to the accounts provided by parents of infants, parents of toddlers framed their descriptions to suggest that responsibility for the child’s consumption of adequate quantity and/or variety of foods is assumed by the parent. Perhaps parents have internalised the child’s food intake as a reflection of their own parenting.

### Aim 2: Mealtime interaction patterns

Three broad themes emerged from the data regarding parents’ descriptions of mealtime interactions (Fig. 1). The first focused on parent agency, in which parents’ accounts indicate that they construct the child’s food consumption as their responsibility (‘parent concern’). The second arose from the construction of child agency, in which parents allow their child’s food preferences to drive food interactions (‘child control’). Finally, parents’ construction of mealtimes as battlegrounds between child resistance and parent concern (‘conflict’) emerged as a dominant theme.

#### Parent concern

Mealtime interactions in the context of fussy eating occurred within a heightened emotional context expressed by parents as frustration, despair and guilt. These emotions coloured parents’ descriptions of mealtime interactions. Parents anticipated ongoing difficult child eating behaviours, based on a history of challenging mealtimes. Parents described feeling ‘at a loss’ and the negative experience of mealtimes as an intractable problem:

‘I think my only concern is how much I can tolerate … because I know, I know it’s going to be hard, and whether or not I have the patience for it.’ (Parent 1; 30-month-old child)

‘Yeah look, I mean you rub your hands in frustration don’t you? I’ve watched him for twelve months not touch anything I’ve made at dinner at night.’ (Parent 2; 48-month-old child)

‘I don’t know what to do. I’m trying everything … I’m lost.’ (Parent 9; 10-month-old child)

Sometimes the frustrations concerning feeding occurred within the context of parents’ unrealistic expectations of child eating, that were intimately tied to perceptions of social norms and judgements:

‘I mean, the bottom line is though, you hate it when they don’t eat, it’s awful. And you know I get frustrated, we have a birthday party for him and we have a barbecue and he’s the only one not eating anything. And you know, people sort of look at you and you think “Oh crikey”.’ (Parent 2; 48-month-old child)

Other parents spoke of expectations based on the child’s feeding history. Foods and textures that were once, but no longer accepted, were used as an indicator of the child’s escalating fussy eating behaviour:

‘He’d just usually, like with fish fingers once a week or twice a week maybe he’d eat three maybe four of those at one sitting and then [he’d] eat his vegies as well but he’s not even eating that. And like fish fingers is something we used to give him if we were, you know, we’d cook them before we went out and put them in a little container and he’d eat them in the car. But no. He won’t even do that.’ (Parent 5; 12-month-old child)

‘But he’s just progressively getting worse ... I started off one solid feed a day and that was basically only the rice cereal … I’m still pureeing everything because he just … I’ve tried to include some lumps … But he just holds it in his mouth and throws up or he gags on it and throws up, one of the two.’ (Parent 9; 10-month-old child)

Interestingly, parents did not describe concern about their child’s weight and in some cases explicitly noted this was not a problem. Evidently, calls focused on the specific feeding problems, even in the absence of weight or growth problems:

‘He doesn’t feel any different physically to look at you know, the way he’s been going. He’s always been fairly tall and slimmish and he just doesn’t look any different to me really.’ (Parent 4; 24-month-old child)

‘So I’m not concerned about the weight because he’s quite a tall child.’ (Parent 5; 12-month-old child)

‘Fine. She’s a big girl for age, she’s quite heavy for her age, solid girl.’ (Parent 10; 12-month-old child)

Concerns about the variety and quantity of foods consumed were pervasive even in the absence of any notable effects on growth or health. Although parents described their child’s weight as unproblematic, mealtime behaviours and the adverse effect of these on parent well-being are patent. Notable in the descriptions are parents’ emotive language and distress. They highlight the intense emotional component of the experience of fussy eating. Concern, for these parents, permeates the mealtime environment.

#### Child control

Across the period of infancy and toddlerhood, children increasingly assume control at mealtimes, expressing their autonomy in food choices and behavioural responses to the mealtime environment. Parents’ accounts of mealtimes suggest that their reaction to their child’s food refusal is filtered through a lens of perceived nutritional shortfall and they respond by supplementing with formula and providing preferred foods. Such reactive responses provided solace that the child was consuming some form of nutrition:

‘And then he didn’t really eat much during the day. And I’ve still got him on his formula bottles because I thought he needs some kind of nutrients.’ (Parent 1; 30-month-old child)

‘… he’s having around three or four bottles a day, close to 200 ml. Um, yeah I’d say he’s probably having more milk than anything else.’ (Parent 5; 12-month-old child)

Emerging from the data is a sequence of events that suggest that fussy eaters make the connection that refusing to eat a certain food will elicit the parent’s provision of a preferred food. For example, one parent described this process in changing from lower- to higher-sugar breakfast cereal:

‘And you do you give them one thing but then suddenly they’ll start refusing to eat it so I’ve sort of moved away from the Weetbix and thought well I’ll try to give him the cereal that he will eat so I’ve given him Milo [chocolate flavoured] cereal.’ (Parent 4; 24-month-old child)

Parents reported providing the child’s preferred foods, even though they clearly evidenced knowledge that these foods were invariably not appropriate or nutritious. Parents’ fear that the child would ‘go hungry’ was ubiquitous and a key mechanism explaining their willingness to provide and tolerate the child’s consumption of ‘wrong foods’:

‘But what I have been doing is giving him either a yoghurt or an ice cream afterwards and I know that’s perhaps wrong because he’s not ate but I’ve always thought, “God well I’ve got to give him something to eat”.’ (Parent 4; 24-month-old child)

‘I do offer but he just doesn’t eat. And then he’ll get a drink. He’ll have some orange or lemonade or he’ll have, I mean he loves coffee. I know he shouldn’t have coffee but …’ (Parent 4; 24-month-old child)

Children’s emerging expressions of their autonomy of eating were presented as a challenge to parents’ intentions to feed:

‘Yeah he won’t eat anything like that. He won’t eat anything he won’t even allow us to attempt to give him anything off a spoon. He just pushes us away.’ (Parent 5; 12-month-old child)

The parent’s description suggests that the behaviour is at odds with the social conceptualisation of a ‘good eater’. The emerging autonomy of the child in expressing food preferences and regulating his/her own type and quantity of intake, while a developmental advance in connecting with food and mealtimes, necessarily changes the control parents have at mealtimes. Emerging tensions and relationship difficulties at mealtimes were underpinned by parent anxiety and, in the cases seeking support on the helpline, presented internal tensions for parents between fear of child hunger and providing poor nutrition. Those calling for help described prioritising satiation over nutritional quality.

#### Conflict

Parents described children’s fussy eating in the context of non-compliance. Conflict arose when both parent and child met with resistance. In response, parents described using a variety of strategies to feed their child, including physically prompting the child to eat or even forcing food on them. However, these attempts often did not result in parents meeting their own needs of feeding their child:

‘Yeah we give it to him but he just throws it on the floor, he’s always done that. He just throws everything on the floor. We can’t seem to stop him from doing it … I used to get him a drink in a cup and he’d stand there and thought it was great and drink out of it but now he just um I’ll go to you know kneel down say come on have a drink of milk, I’ll go to put it to his lips and he pushes it away and doesn’t want us to do anything for him.’ (Parent 5; 12-month-old child)

Two parents described using distraction during mealtimes in order to ‘get’ children to eat:

‘So I have this thing, I have to divert him, I’ve got people standing, like my husband will stand behind me and make all these … oh gosh …’ (Parent 9; 10-month-old child)

‘He’ll eat it if I entertain him, if I get down on my hands and knees and jump around like a crazy woman but he’ll finish what he’s got if we entertain him … And yeah it’s like we have all sorts of toys to entertain him to get the food down him.’ (Parent 11; 7-month-old child)

Conflict was sometimes described as parents forcibly feeding the child:

‘Well, I mean it’s, no no and I mean to be honest he’s, he’s quite headstrong and he doesn’t even like sitting at the table half the time. He wants to be out you know, playing with toys, he doesn’t want to sit at the table but I do make him sit at the table. Um, I’ve even taken lately, which I shouldn’t be doing, when he leaves half his breakfast, I’m following him round with teaspoons of cereal you know … I’m following him to the next room and I managed to shove some food down him while he’s in another room.’ (Parent 4; 24-month-old child)

Parents’ descriptions of the child as ‘bad’ or troublesome in the context of feeding included those suggesting the child must be ‘disciplined’.

‘And it’s just really, really difficult to try and um be totally, you know, disciplined with him … So um, but obviously I’ve been trying my best … I know it sounds like an excuse but I just have trouble keeping everybody out of the fridge.’ (Parent 4; 24-month-old child)

In this example ‘discipline’ related to food access. Notable here is the absence of parent attempts to structure food access and set mealtimes.

The conceptualisation of mealtimes as a battleground was evident in the parents’ wording. ‘Success’ was measured by ‘getting food in’, rather than the teaching process of increasing preferences for nutritional foods. As evidenced by the two quotes above, parents of toddlers resorted to subversive behaviour to feed their child. Their accounts contrast with those provided by parents of infants who evidenced attempts to understand the perspective of the child as a learner:

‘And in the beginning I just thought oh it’s new he doesn’t know … [he’s] trying to get used to it and not sure what he’s doing but it’s continued on and I mean he’s good for the first few minutes of feeding but then it’s almost like he gets bored.’ (Parent 11; 7-month-old child)

Among the parents of toddlers only one parent described avoiding conflict by continuing to offer a variety of foods:

‘No he won’t eat fruit. I mean last week, I got him to eat one piece of watermelon, the first time he’s ever ate it. Umm he used to nibble on an apple but he doesn’t really eat that. I’ve got grapes in, cherries, mango. Bananas … I’ve offered them several times.… and he’ll just lick them, or he, I mean, he used to chew on an apple or chew on a watermelon and then when he crunched it into bits he just spat it back out.’ (Parent 4; 24-month-old child)

With this one exception, the picture presented by parents calling for help was of mealtimes as a battleground rather than one of positive relationships with food and a learning experience in which parents anticipate and are responsive to children’s emerging autonomy to guide their self-regulation.

## Discussion

The current study provides a rich understanding of parents’ perceptions of fussy eating by capturing their accounts at a point of crisis that has prompted a call for support to a nurse-staffed helpline (Child Health Line). In contrast to previous qualitative studies^(^ ^14^ ^,^ ^28^ ^)^ our data provide real-time descriptions of fussy eating. These highlight high levels of emotion evoked in parents as they experience the challenges of their child’s food refusal and associated behavioural difficulties at mealtime. Such rich description of emotion is unlikely to emerge from quantitative data or alternative retrospective qualitative methods, and provides a unique understanding through parent voice.

Parents’ accounts presented in the current study, like previous literature, describe fussy eating as food rejection that limits variety and quantity of food intake^(^ ^6^ ^,^ ^9^ ^,^ ^29^ ^)^. However, new findings are suggestive of a trajectory from a normative developmental transition from milk-based foods into a behavioural and nutritional conflict underpinned by parent concern and child control. The sample, although small, provided a cross-section of infants and toddlers whose parents sought advice regarding feeding of solid foods. These two groups of parents ascribed different meanings to their child’s rejection of foods. Parents of infants calling for help described rejection of foods and/or of diverse textures. They sought advice or reassurance regarding their feeding strategy and framed their problem as a challenge in transitioning their infant from a milk-based diet to a predominantly solid-based diet. The problem of fussy eating was conceptualised as an issue of shared learning for infant and parent. In contrast, parents of toddlers presented their child in dichotomous terms (e.g. ‘will’/‘won’t’), the problem of fussy eating as intractable and the locus of the problem as the child. Parent descriptions evoked ‘future-oriented’ language that presented the child as a ‘bad’ eater and mealtimes as a place of conflict in which they ‘gave in’ and permitted ‘wrong’ foods for fear their child would ‘go hungry’. Nutritional quality of food was secondary to satiation in the parents’ accounts.

Emotional distress, particularly feelings of judgement or failure as a parent were potent in the parent’s accounts. High levels of anxiety about quantity of foods consumed (‘going hungry’) perpetuated a cycle of reactivity in which parents forfeited food quality in favour of the child’s preference, approached mealtimes with a sense of dread and engaged in feeding practices that rewarded inappropriate eating behaviours and poor nutritional intake. A recent study^(^ ^30^ ^)^ similarly found that mothers (*n* 296) of children (71-months-old) who were concerned that their child was undereating were more likely to report their child as a ‘fussy eater’ and pressure them to eat and use bribes. Our data suggest that pressure to eat, subversive feeding and bribes were all used despite the parents’ clear descriptions of their child as healthy weight. However, these findings might be viewed in the context of recent evidence showing that fussy eating^(^ ^31^ ^)^, particularly persistent fussy eating in the early years^(^ ^32^ ^)^, is associated longitudinally with underweight.

Concern about quantity consumed perpetuated provision of foods of poor nutritional value. For example, parents described succumbing to the child’s request for high-sugar foods and ‘giving up’ on providing nutritional foods, particularly vegetables. Such reactive feeding served to limit their child’s exposure to a variety of foods and their opportunity to develop more mature eating patterns. Children generally prefer energy-dense and nutrient-poor foods over vegetables, which is likely genetically influenced^(^ ^33^ ^)^. However, repeated neutral exposure to a variety of vegetables has been shown to increase a child’s preference for vegetables^(^ ^34^ ^,^ ^35^ ^)^. These accounts suggest that parents encountering escalating feeding problems do not understand that food choice and rejection are developmental phenomena associated with emerging child agency and autonomy^(^ ^36^ ^)^. Parents’ responses to the child’s expression of agency in selecting foods may be critical in the aetiology of fussy eating.

Parent understanding of typical developmental transition in early childhood and delineation of division of responsibility^(^ ^19^ ^)^ across this transition may be particularly important in attenuating eating problems. The emergence of conflict and reactive feeding occurred when parents identified their role as ‘getting’ the child to eat rather than providing food, and resulted in parent intrusiveness in the child’s eating including forcible feeding and distraction. Such behaviours are of concern. Distracted eating, for example children eating in front of the television, has been consistently associated with poor quality of food intake^(^ ^37^ ^)^. Moreover, the early use of distraction may disrupt developing self-regulation and attention to cues of hunger and satiety, and detract from parental modelling of healthy eating behaviours^(^ ^38^ ^)^. The effects of such strategies on longer trajectories into problematic eating patterns and weight status warrant further investigation. Education regarding the delineation of responsibility between parent and child, particularly with regard to structuring mealtimes (when and where) and choices of food intake, may be important^(^ ^39^ ^,^ ^40^ ^)^. Our data suggest that such approaches may alleviate parent concern that rewards and perpetuates ongoing fussy eating, a behaviour that, for most children, is transitory^(^ ^5^ ^)^.

### Strengths and limitations

The strengths of the present study lie in the unique, real-time and rich nature of the data. As calls were anonymous, social desirability bias seen in interview or survey methods is likely to be minimal. However, there are several limitations to consider in the interpretation of results. Collection of demographic information was limited and does not allow more detailed description of the sample to assist interpretation of the results. Furthermore, as calls were answered by different nurses across the corpus of calls, call-taker style may have impacted disclosure. The twelve calls analysed here were not sampled but in fact all those relating to fussy eating made to the Child Health Line. This is a small proportion (2 %) of the helpline calls and lower than the prior population estimates^(^ ^10^ ^,^ ^13^ ^)^. These data represent only those parents who reached a point of crisis and sought helpline support. Other sources of support may be sought and accessed prior to point of crisis. Although detailing an endpoint, we therefore cannot claim population representation. Of the parent callers, 50 % were from high socio-economic areas as indicated by SEIFA coding of their postcode^(^ ^26^ ^)^. The extent to which feeding problems occur across different socio-economic strata cannot be accurately assessed from this sample but warrants further investigation to identify sources of support. Moreover, research is required in socio-economically disadvantaged areas where higher rates of feeding problems have been identified^(^ ^5^ ^)^.

## Conclusion

Parents’ real-time descriptions of their young child’s eating behaviours at a time of crisis capture the highly charged emotional underpinnings of mealtime interactions associated with fussy eating. Importantly, they show the child’s emerging assertion of food autonomy can escalate parents’ emotional distress that, in the short term, initiates reactive patterns of feeding and, potentially, ongoing patterns of poor dietary intake. The work identifies the importance of educational support across the transition from a milk-based to a solid-food diet. Educational and emotional support is particularly important for parents whose child has greater challenges across this transition.
